# Safranal, a Saffron Constituent, Attenuates Retinal Degeneration in P23H Rats

**DOI:** 10.1371/journal.pone.0043074

**Published:** 2012-08-10

**Authors:** Laura Fernández-Sánchez, Pedro Lax, Gema Esquiva, José Martín-Nieto, Isabel Pinilla, Nicolás Cuenca

**Affiliations:** 1 Department of Physiology, Genetics and Microbiology, University of Alicante, Alicante, Spain; 2 Department of Ophthalmology, University Hospital Lozano Blesa, Zaragoza, Spain; University of Florida, United States of America

## Abstract

Saffron, an extract from *Crocus sativus*, has been largely used in traditional medicine for its antiapoptotic and anticarcinogenic properties. In this work, we investigate the effects of safranal, a component of saffron stigmas, in attenuating retinal degeneration in the P23H rat model of autosomal dominant retinitis pigmentosa. We demonstrate that administration of safranal to homozygous P23H line-3 rats preserves both photoreceptor morphology and number. Electroretinographic recordings showed higher a- and b-wave amplitudes under both photopic and scotopic conditions in safranal-treated versus non-treated animals. Furthermore, the capillary network in safranal-treated animals was preserved, unlike that found in untreated animals. Our findings indicate that dietary supplementation with safranal slows photoreceptor cell degeneration and ameliorates the loss of retinal function and vascular network disruption in P23H rats. This work also suggests that safranal could be potentially useful to retard retinal degeneration in patients with retinitis pigmentosa.

## Introduction

Retinitis pigmentosa (RP) refers to a heterogeneous group of inherited neurodegenerative retinal disorders that cause progressive peripheral vision loss and poor night vision, which eventually leads to central vision impairment. RP has been related to more than 100 different mutations of the rhodopsin-encoding gene (*RHO*), which altogether account for 30–40% of autosomal dominant cases. The P23H mutation of this gene is the most prevalent cause of RP [1]. In the United States, this mutation alone accounts for about 12% of autosomal dominant RP cases [2]. The majority of RP-causing mutations in the *RHO* gene, including P23H, cause misfolding and retention of rhodopsin in the endoplasmic reticulum of transfected cultured cells [3]. These studies also suggest that the RP mechanism may involve a cellular stress response [4] resulting in programmed photoreceptor cell death or apoptosis, a final common pathway for different retinal diseases [5].

No effective therapy has been found for RP. It would be thus interesting to address potential treatments that would at least delay the progression of the disease. Recent works have suggested that supplementation with antioxidants may help delay or even prevent retinal degeneration associated with RP. Antioxidants have been shown to be effective in preventing retinal degeneration in mouse models of RP [6]. These studies have shown that a mixture of antioxidants (alpha-tocopherol, ascorbic acid and alpha-lipoic acid) promotes cone survival in rd1 mice. The same mixture of antioxidants slows down rod degeneration in rd10 mice [7]. On the other hand, it has been reported that a mixture of lutein, zeaxanthin, glutathione and alpha-lipoic acid is able to slow the death of photoreceptors in rd1 mice [8], [9].

The pistil of *Crocus sativus*, commonly known as saffron, has been commonly used in traditional medicine as an anodyne and sedative. In the retina, saffron is considered to help blood circulation, cures macula lutea and retinopathy ischemic caused by old age [10]. Modern pharmacological studies have demonstrated that saffron and its constituents protects against damage, exerting anti-ischemic [11], [12], [13], [14], anticonvulsant [15], anxiolytic [16], [17], antidepressant [18], [19], anti-inflammatory [20], hypotensive [21], [22] and antitumor [23], [24], [25], [26] properties.

Safranal (2,6,6-trimethyl-1,3-cyclohexadiene-1-carboxaldehyde), the main component of essential oil of saffron, exhibits antioxidant activity [27], [28], [29] and has the ability to bind and stabilize the DNA molecule [29], [30]. *In vitro* studies have demonstrated that it is capable of neutralizing free radicals [27], [29]. *In vivo*, it is capable of suppressing the genotoxicity caused by methyl methanesulfonate [31], [32]. In ischemic rats, safranal also exerts a protective activity against oxidative damage in skeletal muscle [13] and cerebral tissues [12], [14], and has also been reported to have anticonvulsant activity in mice with chronic attacks, having been shown to reduce the duration of attacks, delay tonic stages and protect mice from death [15]. Diet supplementation with saffron extract protects photoreceptors from death by exposure to bright light [33] and improves retinal function in early age-related macular degeneration [34]. All these data suggest that safranal is a powerful antioxidant that fights oxidative stress in neurons [35]. However, to date, the effects of this compound on experimental models of degenerative retinal hereditary diseases have not been studied.

The aim of this study was to evaluate the effectiveness of safranal as a neuroprotective agent on homozygous P23H line-3 rats. To do so, we used functional (ERG) and morphological (histological labeling) techniques. The capacity of safranal to prevent the loss of synaptic contacts in the outer plexiform layer (OPL) was also evaluated. If safranal is shown to have positive effects in this animal model, this could potentially lead to its preventive use in patients affected by RP.

**Figure 1:**
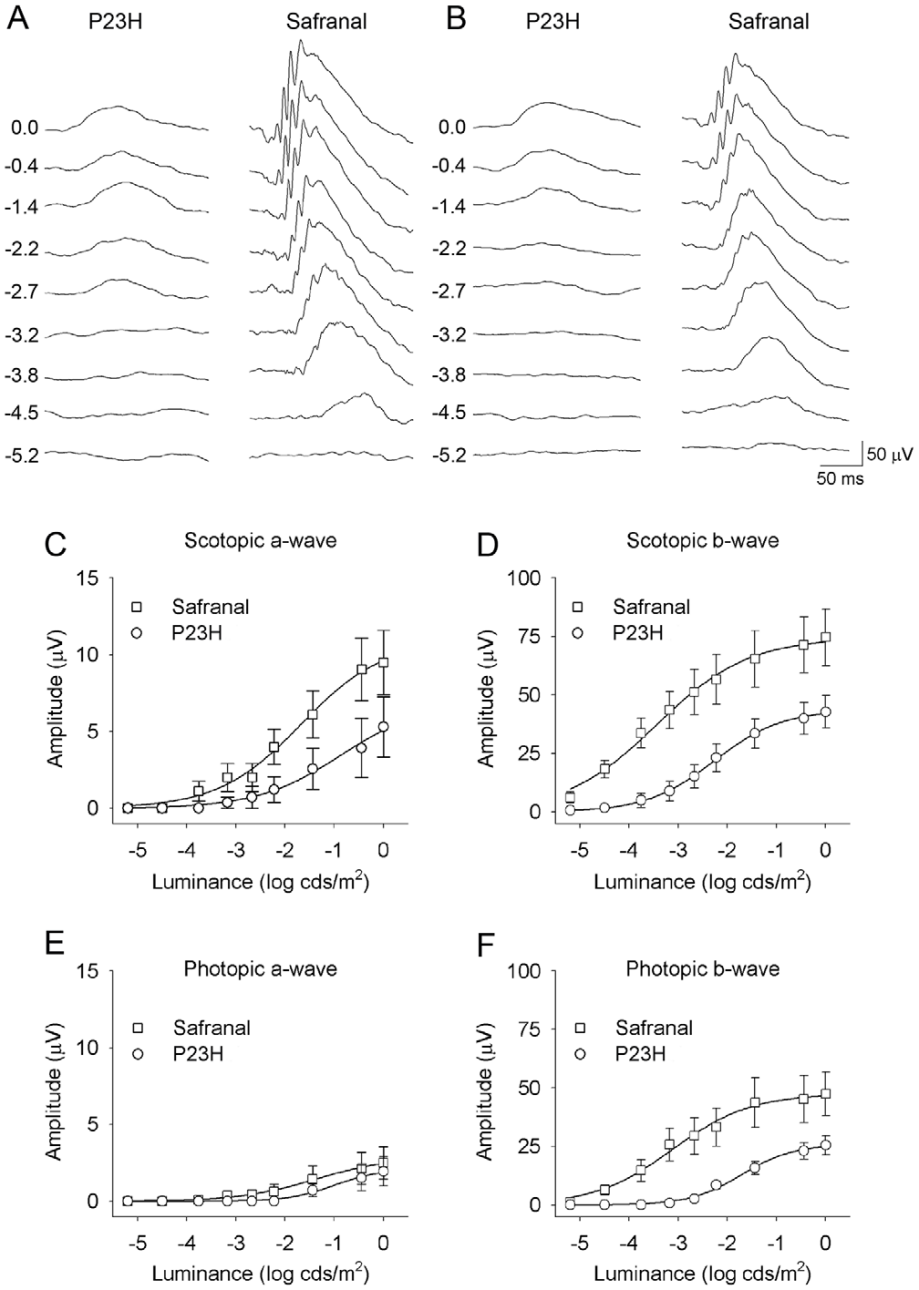
Retinal function in control and safranal-treated P23H rats. (A–B) Example of scotopic (A) and photopic (B) ERG traces from a P120 rat treated with vehicle (left) or safranal (right). Units on the left of panels represent the luminance of the flashes in log cd·s/m^2^. (C–D) Stimulus intensity curves for mixed scotopic a-waves (C) and b-waves (D) from rats administered safranal (squares) or vehicle (circles). (E–F) Intensity response of photopic a-waves (E) and b-waves (F).

**Figure 2:**
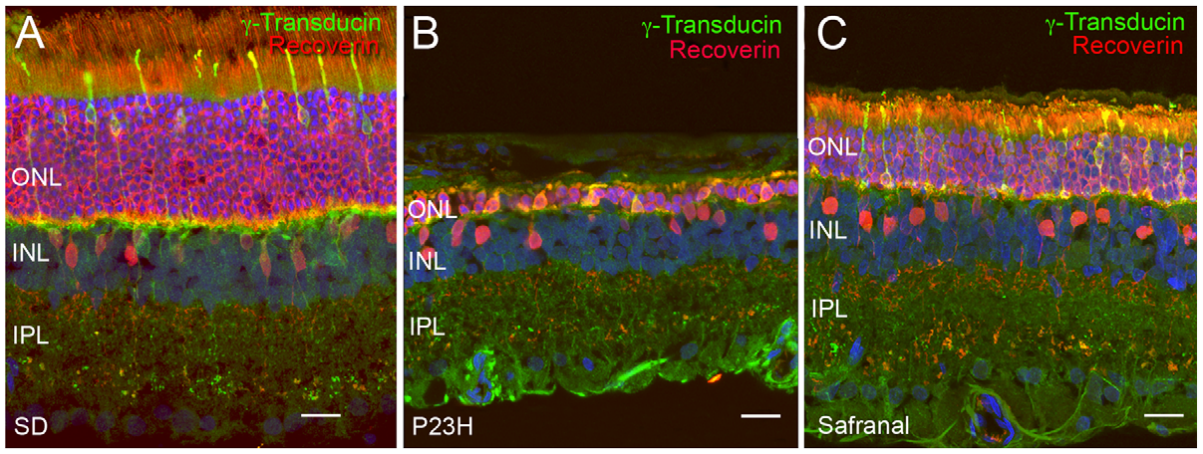
Photoreceptor cell bodies number. Representative retinal section stained with TO-PRO (blue), γ-transducin (green) and recoverin (red) from a wild-type animal (Sprague Dawley, SD, A), a P23H rat treated with vehicle (B) and a P23H rat treated with safranal (C). All images were collected from the central area of the retina, close to the optic nerve. Note that the number of photoreceptor rows in the vehicle-treated P23H rat is low (B), as compared to those present in the retina of the safranal-treated P23H animal (C). ONL: outer nuclear layer, INL: inner nuclear layer, IPL: inner plexiform layer. Scale bar: 20 μm.

**Figure 3:**
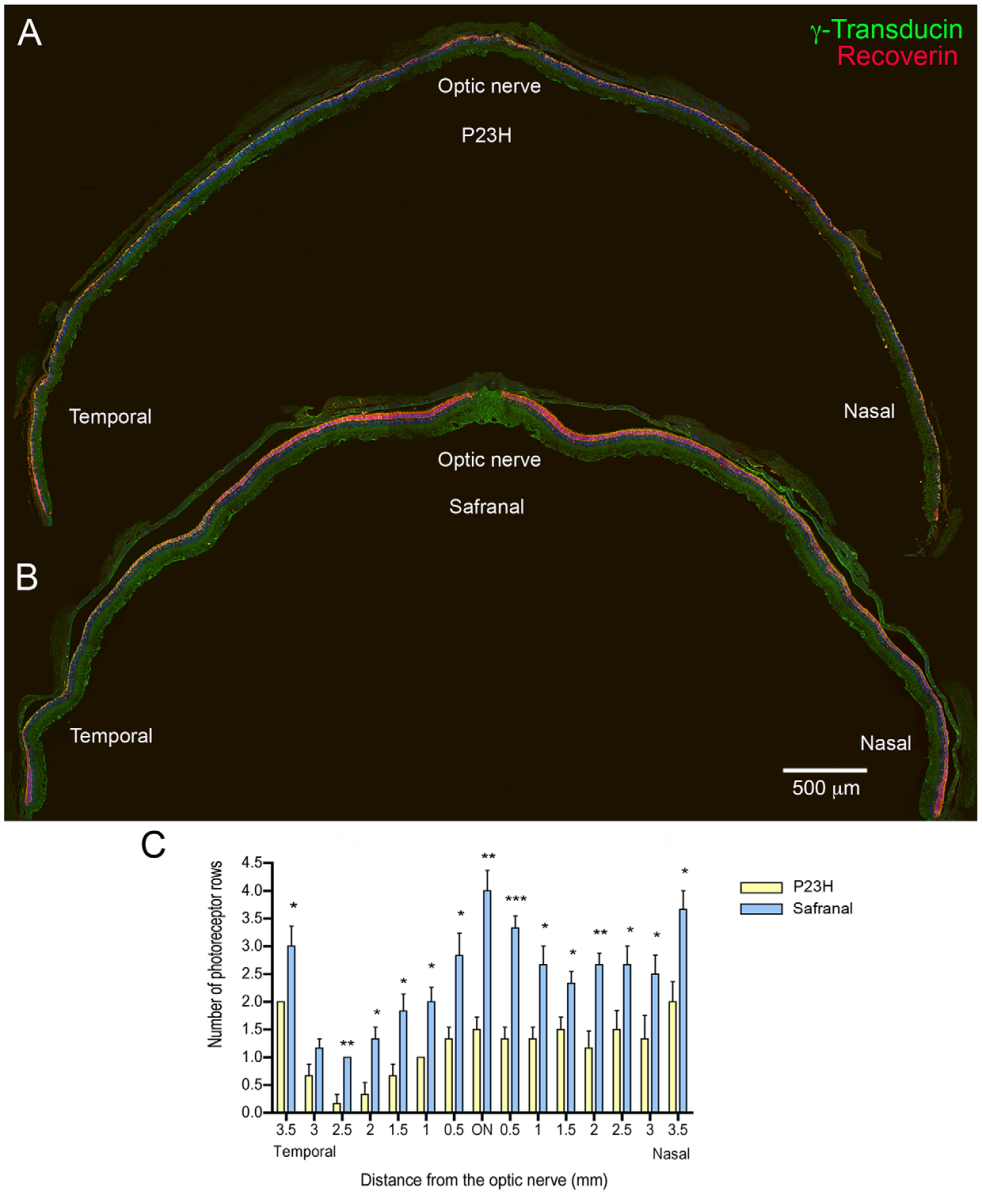
Assessment of photoreceptor rows in control and safranal-treated P23H rats. (A–B) Vertical sections from the temporal to nasal area of the retina at the optic nerve level in P23H rats administered with vehicle (A) or safranal (B), stained with TO-PRO (blue), γ-transducin (green) and recoverin (red). (C) Quantification of the number of rows at the ONL along retinal sections (measured at 0.5 mm intervals) in control and safranal-administered P23H animals (n = 6 in both cases). * *P*<0.05, ** *P*<0.01, *** *P*<0.001; Student's *t*-test.

**Figure 4:**
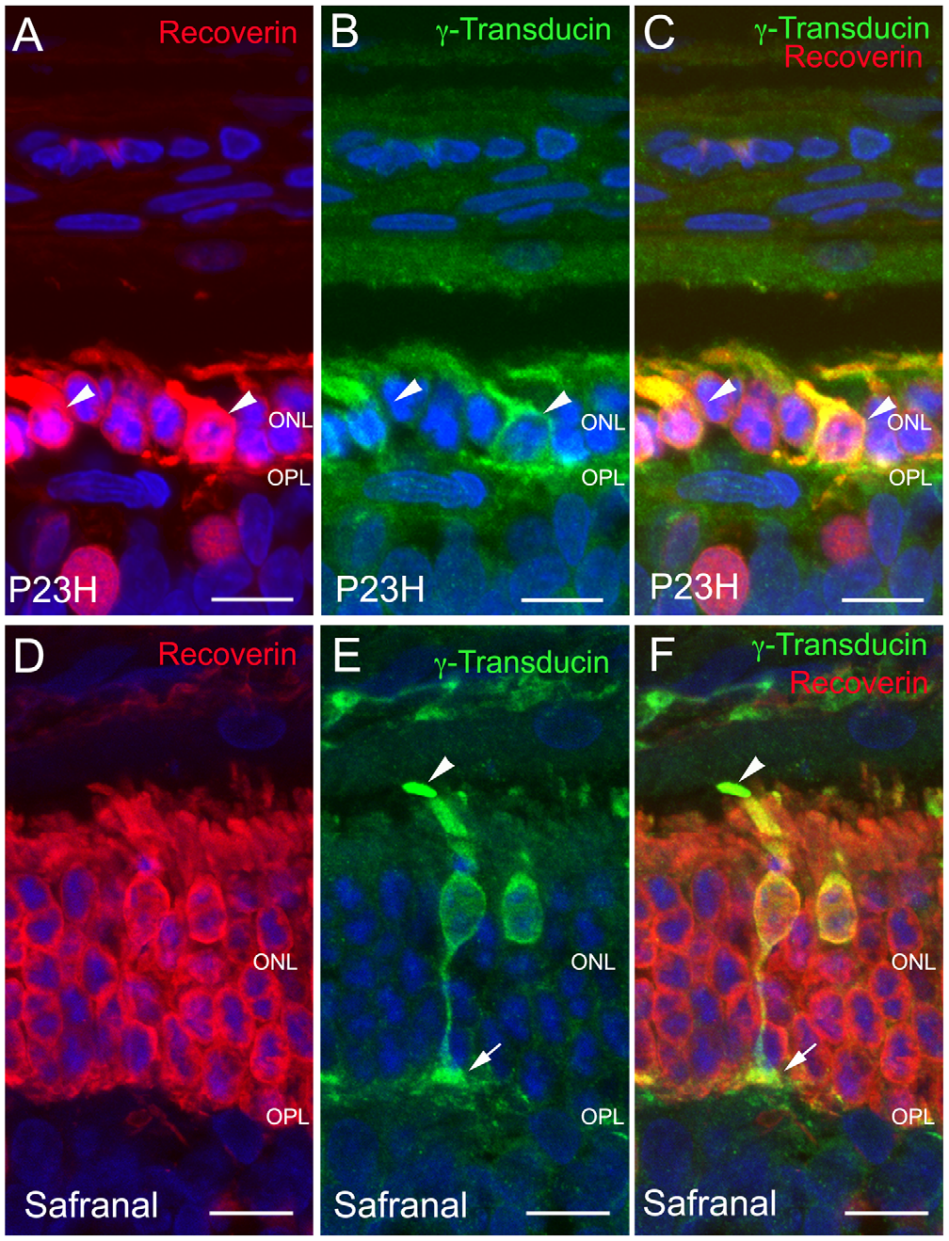
Photoreceptor morphology in control and safranal-treated P23H animals. Vertical sections of retinas from P23H rats treated with vehicle (A–C) or safranal (D–F). Nuclei stained with TO-PRO (blue). (A, D) recoverin (red) stained retinas showing a more profuse degeneration in control animals (A) than that observed in safranal-treated rats (D). (B, E) Cone specific staining with γ-transducin (green) showing smaller cell size and shorter and swollen outer segments in control animals (B, arrowheads) as compared to safranal-treated rats (E) where all cone structures from outer segment (E, arrowhead) to pedicle (E, arrow) can be observed. (C, F) Double immunolabeling for recoverin and γ-transducin. All images were collected from the central area of the retina, close to the optic nerve. ONL: outer nuclear layer, OPL: outer plexiform layer. Scale bar: 10 μm.

**Figure 5:**
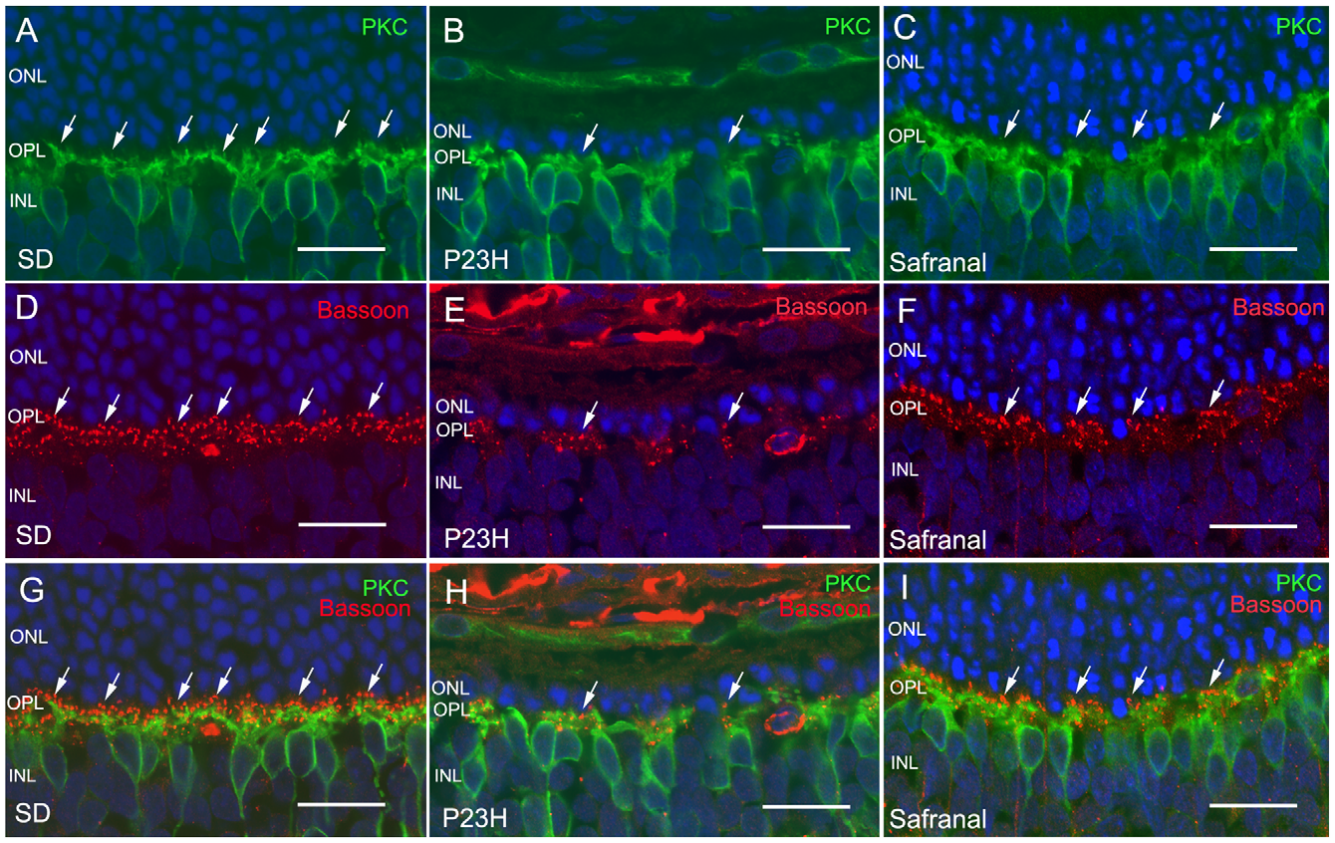
ON rod bipolar cells and their synaptic connectivity. Immunolabeling of retinal vertical sections from wild-type rats (Sprague Dawley, SD) (A, D, G) and P23H animals treated with vehicle (B, E, H) or safranal (C, F, I). Nuclei stained with TO-PRO (blue). (A–C) Staining of retinal ON rod bipolar cells with PKC (green). Note that cell bodies and dendrites were preserved by safranal. (D–F) Labeling of photorreceptor synaptic ribbons with antibodies against bassoon (red). (G–I) Double immunolabeling for PKC and bassoon, showing the preservation by safranal of synaptic contacts (arrows) between photoreceptors and bipolar cells. All images were collected from the central area of the retina, close to the optic nerve. ONL: outer nuclear layer, OPL: outer plexiform layer, INL inner nuclear layer. Scale bar: 20 μm.

**Figure 6:**
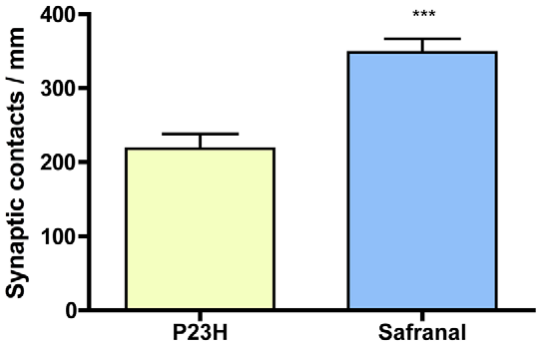
Synaptic contacts in OPL. Quantification of the number of photorreceptor synaptic ribbons at the OPL along retinal sections in control and safranal-administered P23H animals (n = 8 and n = 6, respectively in). *** *P*<0.001; Student's *t*-test.

**Figure 7:**
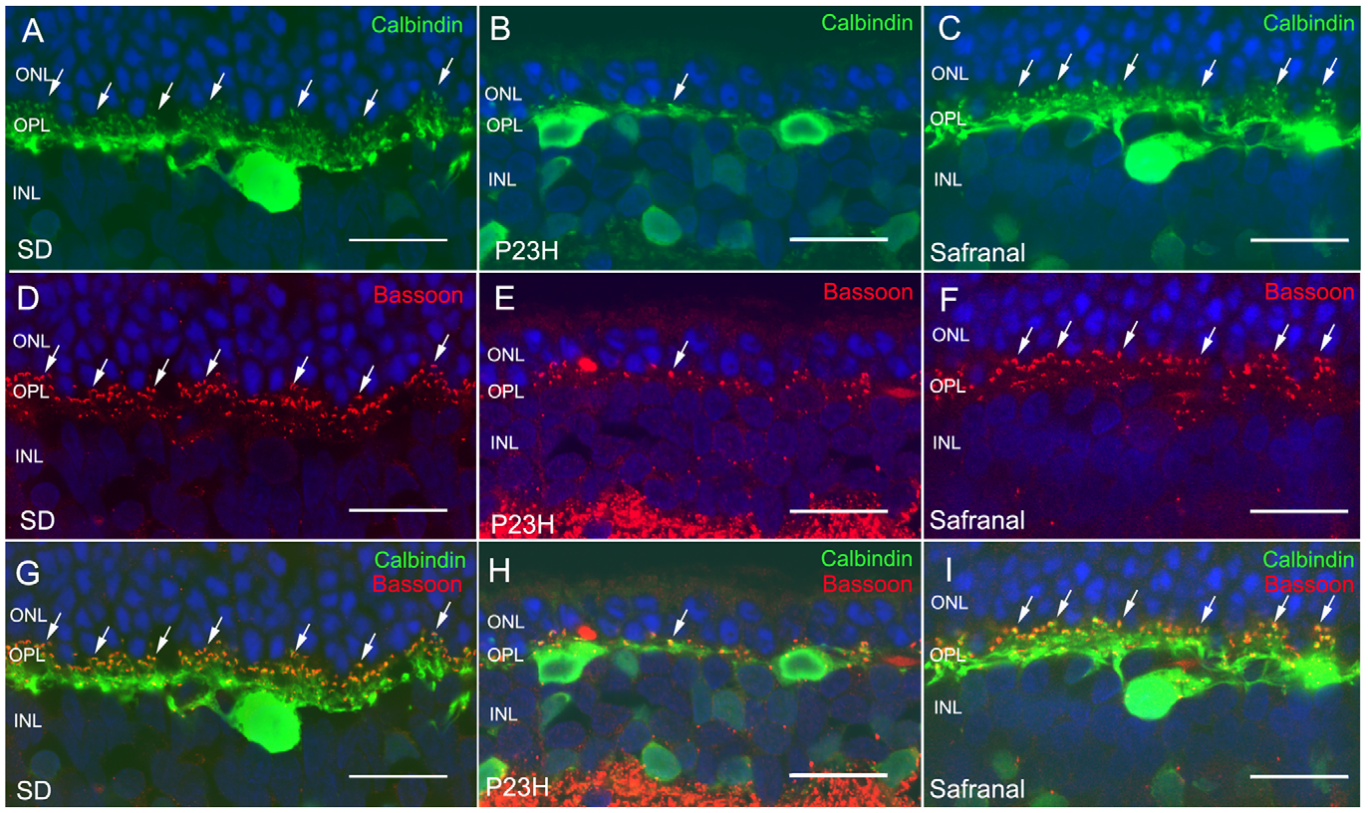
Horizontal cells and their synaptic connectivity. Vertical sections of retinas from wild-type rats (Sprague Dawley, SD) (A, D, G) and P23H animals treated with vehicle (B, E, H) or safranal (C, F, I). (A–C) Horizontal cells labeled with antibodies against calbindin. Note that the number of horizontal cell terminals in safranal-treated rats was higher than in vehicle-treated animals. (D–F) Labeling of photorreceptor synaptic ribbons with antibodies against bassoon (red). (G–I) Double immunolabeling for calbindin and bassoon showing a larger number of synaptic contacts (arrows) between photoreceptor and horizontal cells in safranal-treated rats (H) than observed in the control rats (I). All images were collected from the central area of the retina, close to the optic nerve. ONL: outer nuclear layer, OPL: outer plexiform layer, INL inner nuclear layer. Scale bar: 20 μm.

**Figure 8:**
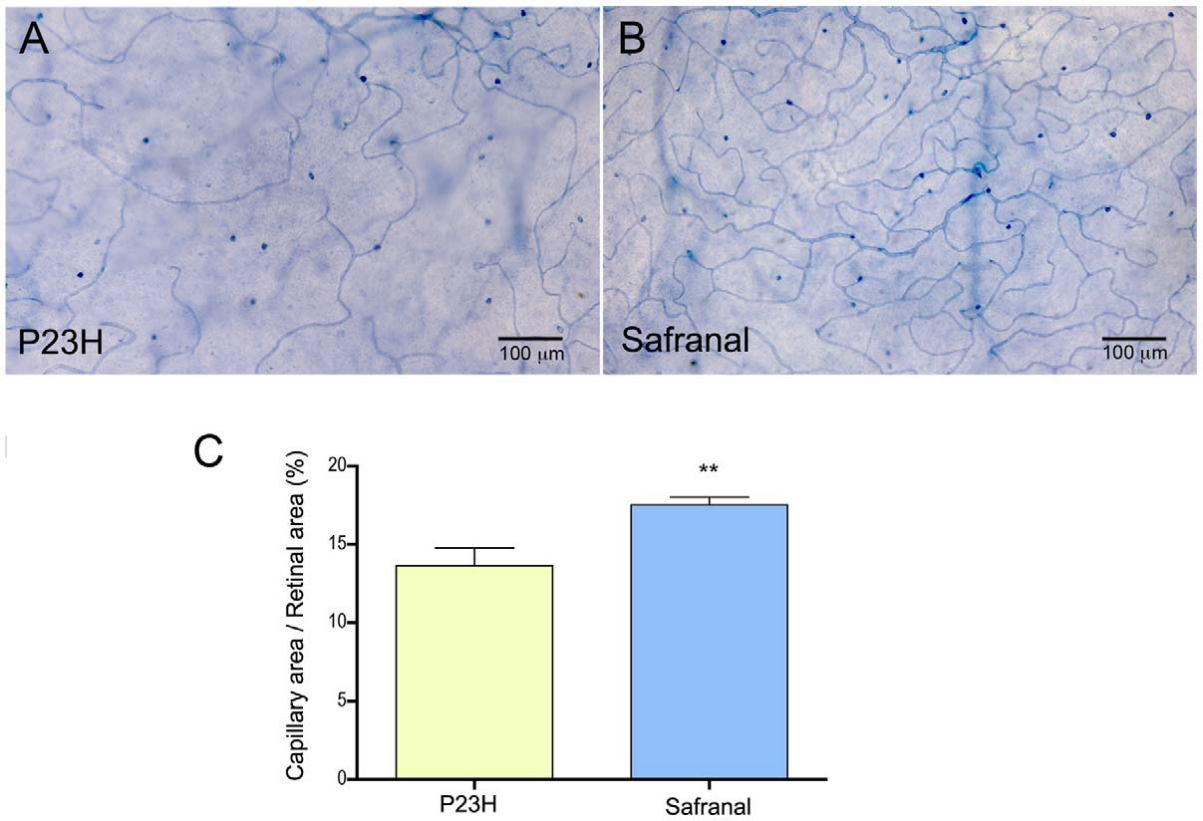
Retinal capillary network. (A–B) Whole-mount retinas from P23H rats treated with vehicle (A) or safranal (B), stained with NAPDH diaphorase. Note that the retinal capillary network was more extensive, with more capillary loops in safranal-treated animals. (C) Measurements of the relative capillary density (capillary area/retinal area) showing higher values in safranal-treated rats than in control animals (n = 6 and n = 3, respectively). ** *P*<0.01; Student's *t*-test.

**Table 1:** Primary antibodies.

Molecular marker	Antibody	Source	Dilution
Calbindin D-28K	Rabbit polyclonal	Swant	1∶500
Protein kinase C, alpha isoform	Rabbit polyclonal	Santa Cruz Biotechnology	1∶100
Bassoon	Mouse monoclonal	Stressgen	1∶1000
Recoverin	Mouse monoclonal	J.F. McGinnis, University of Oklahoma	1∶2000
Transducin, Gαc subunit	Rabbit polyclonal	Cytosignal	1∶200

## Results

### Safranal preserves retinal function

To determine whether safranal was able to preserve photoreceptor function in P23H rats, we performed scotopic and photopic flash-induced ERGs in vehicle- and safranal-treated animals at P120. Figure 1 shows that ERG responses were much less deteriorated in rats treated with safranal (P21 to P120) as compared to those in control animals. Under scotopic conditions, the maximum amplitudes recorded for a- and b-waves were 79% and 74% higher, respectively, in safranal-treated animals than those recorded in control animals (ANOVA, *P*<0.05 for scotopic a-waves; *n* = 18 and *n* = 14, respectively; *P*<0.05 for scotopic b-waves *n* = 22 and *n* = 16, respectively) (Fig. 1A, C, D). Similar differences (86%) were observed in the maximum amplitudes of photopic b-waves (ANOVA, *P*<0.05; n = 18 and n = 12, respectively) (Fig. 1B, F). Maximum amplitudes recorded for photopic a-waves were higher in safranal-treated animals as compared to those measured in control animals, although the differences were not significant (Fig. 1B, E). In animals treated with safranal, the thresholds were lower than those in control rats, for both scotopic b-waves (−5.2 log cd·s/m^2^ *vs*. −3.1 log cd·s/m^2^) and photopic b-waves (−4.1 log cd·s/m^2^ *vs*. −1.9 log cd·s/m^2^) (Fig. 1D, F).

### Safranal slows photoreceptor degeneration

To determine whether safranal treatment protects against the degeneration of photoreceptors, we quantified the photoreceptor rows present in the ONL at P120 using the nuclear dye TO-PRO-3. Figure 2 shows a retinal section from a wild-type animal, a P23H rat treated with vehicle, and a P23H rat treated with safranal (P21 to P120). Few rows of photoreceptor cell bodies could be observed in the vehicle-treated ONL (Fig. 2B), as compared to those present in the retina of safranal-treated P23H animals (Fig. 2C). Because retinal degeneration in control P23H rats was heterogeneous, we opted to study the effects of safranal in different areas of the retina, from the temporal to nasal zones. We found that ONL thickness was greater in treated than in control animals in all examined areas (Student's *t*-test; Fig. 3). Safranal showed its strongest neuroprotective effect at the ONL level in the central area of the retina (Fig. 3C). In this area, 4-month-old untreated P23H rats showed 1 to 2 rows of photoreceptor cell bodies (1.5±0.2), whereas treated animals showed 3 to 5 photoreceptor rows remaining (4.0±0.4).

### Safranal helps maintain photoreceptor morphology

To assess whether safranal-treatment had a positive effect on the morphology of photoreceptors, we examined the staining pattern of antibodies against γ-transducin, a specific marker for cones [36], [37], and recoverin, a marker for rods, cones and two bipolar cell subtypes. Longer inner and outer rod segments were observed for safranal-treated P23H rats than for vehicle-treated animals (Fig. 4), where rod degeneration was evident to a greater degree. Even more drastic changes with age were observed in the cone photoreceptors of vehicle-treated P23H rats. At P120, their outer segments were both short and swollen and very small in size (Fig. 4B, C). The axons were also absent and pedicles emerged directly from the cone cell bodies. The exact opposite occurred in safranal-treated animals, where the outer segments, axon and pedicles (Fig. 4E, F; arrows), and typical cone shape were preserved. Additional images of sections are showed as supporting information (Fig. S1).

### Safranal preserves bipolar cell dendrites and their synaptic contacts with photoreceptors

Retinal ON rod bipolar cells are labeled with antibodies against the α isoforms of protein kinase C (PKC). In rat retinas, dendritic terminals of ON rod bipolar cells establish connections with rod spherules through a large dendritic arbor in the OPL (Fig. 5A). In the retinas of vehicle-treated P23H rats, rod bipolar cells at P120 showed few cell bodies and a retraction of their dendrites (Fig. 5B). Dendritic branches were scarce, and some cells had virtually no dendrites whatsoever. Moreover, immunopositive cell bodies were not aligned in the orderly fashion found in wild-type rats. By contrast, in P23H safranal-treated animals, bipolar cell dendrites were preserved and there was a greater number of cell bodies (Fig. 5C).

We then proceeded to study whether the protective action of safranal on the rod bipolar cells was accompanied by the preservation of their synaptic terminals and connectivity in the outer plexiform layer. To determine this, retinal sections were labeled with antibodies against bassoon, a protein constituent of synaptic ribbons present in both rod spherules and cone pedicles in the OPL. Typical bassoon-immunoreactive spots could be observed in retinas of both wild-type and P23H animals (Fig. 5D, E; arrows), but safranal-treated P23H animals showed more bassoon-immunoreactive puncta (Fig. 5E) than control P23H rats (Fig. 5F). The mean number of photorreceptor synaptic ribbons at the OPL resulted significantly higher in safranal-administered P23H animals that measured in control animals (Student's *t*-test, *P*<0.001, n = 6 and n = 8, respectively; Fig. 6). This would indicate that the presynaptic contact elements between photoreceptors and bipolar or horizontal cells were at least partially preserved.

Double immunostaining for bassoon and PKC revealed the relationship between rod photoreceptor axon terminals and bipolar cell dendritic tips. In retinas from vehicle-treated P23H rats labeled at P120 with antibodies against these two markers, few bassoon-positive dots (Fig. 5H; red, arrows) could be seen paired with PKC-labeled bipolar cell dendrites (green). However, in safranal-treated retinas, the number of bassoon-immunoreactive spots associated with bipolar cell dendritic tips was clearly higher (Fig. 5I).

### Safranal preserves horizontal cell dendrites and their synaptic contacts with photoreceptors

Horizontal cell bodies are labeled with antibodies against calbindin. In the retina, these cells are located on the outermost inner nuclear layer (INL) and establish connections with both rod and cone photoreceptors. In wild-type rats, calbindin labeling revealed a punctate staining of dendritic arborization protruding from horizontal cell bodies and connecting with cone axon terminals, together with thin tangential axonal elongations in the OPL, ending in an extensive arborization, connecting to the rods (Fig. 7A). In vehicle-treated P23H rats at P120, a retraction and loss of horizontal cell dendritic tips was observed alongside a decrease in TO-PRO-3-stained photoreceptor rows (Fig. 7B). In contrast, in safranal-treated rat retinas, a higher number of horizontal cell terminals could be observed (Fig. 7C). A double labeling with antibodies against bassoon and calbindin revealed numerous pairings of photoreceptor axons and horizontal cell terminals in safranal-treated P23H animals (Fig. 7I, arrows), in contrast to the relatively fewer contacts observed in vehicle-treated P23H rats (Fig. 7H). This was indicative of the effect that safranal has on preserving synaptic contacts between photoreceptors and horizontal cells.

### Safranal preserves the retinal capillary network

In RP, photoreceptor cell loss is associated with subsequent atrophy of the retinal capillary network. NAPDH diaphorase histochemistry was performed to visualize the retinal vascular network and evaluate whether safranal-treatment was able to prevent the loss of retinal capillaries. As we can see in Figure 8A, P23H rats showed a poor retinal capillary network, in which capillary loops appeared degenerated. In contrast, the capillary network in safranal-treated animals was more extensive, with well-preserved capillary loops (Fig. 8B). Measurements of the relative capillary density (capillary area/retinal area) showed significantly higher values in safranal-treated animals, as compared to those obtained in control animals (Student's *t*-test, *P*<0.01; Fig. 8C).

## Discussion

Our study revealed that systemic treatment with safranal, a constituent of saffron (*Crocus sativus*), is capable of preserving retinal structure and function in homozygous P23H transgenic rats. Previous studies have shown that safranal exerts cytoprotective effects in a wide spectrum of tissues [13], [31], [38], including the nervous system [12], [15], [17], [35]. Moreover, it has been demonstrated that saffron extracts protect against ocular degenerative disorders caused by exposure to bright light [33] or age-related macular degeneration [34]. In this work, we have analyzed the effects of safranal on a rat model of autosomal dominant RP characterized by slow-pace retinal degeneration. We have focused not only on photoreceptor morphology and function, but also on safranal's secondary effects on photoreceptor connectivity, the structure of inner retinal cell layers and capillary network condition.

Transgenic P23H albino rats have been bred to mimic the RP most commonly found in human populations [1], [2]. These rats develop a progressive photoreceptor dysfunction, which is generally consistent with the clinical findings reported for human P23H RP patients [39], [40]. In this animal model, the loss of photoreceptors is accompanied by degeneration of the inner retina [41], which includes a substantial degeneration of retinal ganglion cells [42], [43]. P23H rats retain vision for a relatively long period of their lives, as described for P23H humans, who exhibit significantly better visual acuity and greater ERG amplitudes than patients who are affected by other RP mutations [39], [44]. The slow retinal degeneration that occurs in P23H line 3 rats makes this animal model better suited to the study of the disease in humans than other P23H lines and genetic mouse models, thus giving our results additional clinical relevance. In our experiments, safranal was administered from P21 to P120, when vehicle-treated animals can be considered to have suffered from extensive retinal degeneration [45], [46].

In this study, we found that safranal treatments ameliorated the loss of both rods and cones in P23H rats, and preserved their morphology, as evidenced by specific immunostaining of both photoreceptor cell types. These effects were consistent with the higher amplitudes of both scotopic and photopic responses found in safranal-treated animals as compared to control animals. Both cone and rod structure and function were preserved to a similar degree, as evidenced by the analogous effects found on scotopic and photopic ERG recordings. All these results agree with the findings of a previous study in which the neuroprotective effects of tauroursodeoxycholic acid (TUDCA) were evaluated in P23H rats [46]. The results also echo the results of previous studies, which show that saffron extracts may protect photoreceptors from damaging light, maintaining both their morphology and function.

In addition to the positive preventive effects of safranal on photoreceptor number, morphology and function, P23H safranal-treated rats experienced improved connectivity between photoreceptors and their postsynaptic neurons: horizontal and bipolar cells. Both presynaptic and postsynaptic elements, as well as synaptic contacts between photoreceptors and bipolar or horizontal cells, were preserved in safranal-treated P23H rats. Furthermore, the number of both rod cell bodies and the density of bipolar and horizontal dendritic terminals were higher than in vehicle-treated rats. These results indicate that the safranal effect on retinal morphology and function extends not only to photoreceptors, but also to other retinal cell types. Another interesting possibility is that the preservation of the photoreceptor population prevents the occurrence of secondary degenerative changes in their postsynaptic neurons, thereby preventing the remodeling of the entire retinal circuitry [47].

Previous works have demonstrated that, in the case of RP, photoreceptor cell loss is associated with subsequent atrophy of the retinal capillary network [48] and lower retinal blood flow [49]. In our results, P23H rats showed a poor retinal capillary network, in which capillary loops appear to have degenerated. In contrast, P23H safranal-treated rats showed more extensive capillary networks and better-preserved capillary loops as compared to the control animals. This suggests a positive effect of this compound, not only in preserving both the outer and inner retinal layers, but also in retarding capillary degeneration.

It has been proposed that the cytoprotective effects of safranal are exerted through antioxidative actions [27], [28], [29]. Antioxidants play an important role in health by protecting cells and tissues from the damaging effects of free radicals and singlet oxygen. The retina is one of the tissues with the highest rates of oxygen consumption and the highest energy demand. In this context, cell degeneration causes an energy deficit, leading to an increase of reactive oxygen species (ROS) levels, and an abnormal elevation of cytosolic Ca^2+^ [50]. The antioxidant activity of safranal can also protect DNA and tRNA in the form of ligand-polynucleotide complexes from harmful chemical reactions [29]. Previous studies have shown the protective effect of safranal against DNA damage in the organs of mice [31], [32].

ROS and oxidative stress may cause apoptosis [51], [52] through mechanisms both dependent on and independent of caspase. In this sense, it has been demonstrated that saffron extracts are able to block neuronal cell death induced by both internal and external apoptotic stimuli [53], [54]. In a previous study performed on rats suffering from a myocardial ischemia-reperfusion injury, safranal exhibited a strong antiapoptotic potential, as evidenced by downregulating Bax and caspase3 expression [55]. Additionally, saffron extracts were proved to reduce apoptosis in photoreceptors isolated in primary retinal cell cultures and exposed to damaging blue light [56]. The antiapoptotic characteristic of saffron components makes them interesting candidates for the treatment of retinal neurodegenerative disease.

Currently there is no effective therapy available to halt the evolution of RP or to restore vision once it has been lost. Despite the use of therapies aimed at curbing cell death, the loss of photoreceptors in terms of number and function usually leads to a dramatic remodeling of retinal circuits, which would probably further compromise the transmission of visual information [41]. In this context, the use of therapies like safranal, effective not only in preventing the loss of photoreceptors, but also in slowing the degeneration of inner retinal layers and the capillary network, may be especially interesting, in combination with other therapies based on the implantation of new photoreceptors and anti-inflammatory agents, among others.

## Methods

### Animals and treatments

Homozygous P23H line-3 albino rats, obtained from Dr. M. LaVail (UCSF School of Medicine; http://www.ucsfeye.net/mlavailRDratmodels.shtml), were used as subjects for this study. All animals originated from a colony bred at the Universidad de Alicante. They were housed under controlled humidity (60%), temperature (23±1°C) and photoperiod (LD 12∶12) conditions. Current regulations for the use of laboratory animals (NIH, ARVO and the European Directive 86/609/EEC) were observed to ensure minimal suffering and numbers required for experimentation. The study had the approval of the Research Ethics Committee of the University of Alicante. Safranal (17306, Fluka Chemie AG, Switzerland) was administered to P23H rats at 400 mg/kg (i.p.) twice a week from P21 to P120. Control animals received the same volume of saline at the same experimental times. In order to adjust the amount of safranal administered, each animal's body weight was measured prior to injecting the drug.

### ERG recordings

Following overnight adaptation to darkness, animals were prepared for bilateral ERG recording under dim red light. Animals were anesthetized by injection (i.p.) of a ketamine (100 mg/kg) plus xylazine (4 mg/kg) solution, and kept on a heating pad at 38°C. Their pupils were dilated by topical application of 1% tropicamide (Alcon Cusí, Barcelona, Spain), and a drop of Viscotears 0.2% polyacrylic acid carbomer (Novartis, Barcelona, Spain) was instilled on the cornea to prevent dehydration and to allow electrical contact with the recording electrodes. The electrodes used were DTL fiber electrodes with an X-Static silver-coated nylon conductive yarn, from Sauquoit Industries (Scranton, PA, USA). A 25-gauge platinum needle inserted under the scalp between both eyes served as the reference electrode. A gold electrode was placed in the mouth for grounding purposes. Anesthetized animals were placed on a Faraday cage and all experiments were performed in complete darkness. Scotopic flash-induced ERG responses were recorded for both eyes in response to light stimuli produced by a Ganzfeld stimulator. Light stimuli were presented for 10 ms at 9 different increasing intensities (ranging from −5.2 to 0 log cd·s/m^−2^). Three to ten consecutive recordings were averaged for each light stimulus. A 10 s interval between flashes was used for dim flashes, and up to 20 s for those of the highest intensity. Photopic responses were obtained after light adaptation at 10 cd/m^2^ for 20 min, and stimuli were the same as those under scotopic conditions. ERG signals were amplified and band-pass filtered (1–1000 Hz, without notch filtering) using a DAM50 data acquisition board (World Precision Instruments, Aston, UK). Stimulus presentation and data acquisition (4 kHz) were performed using a PowerLab system (ADInstruments, Oxfordshire, UK). Recordings were saved on a computer for later analysis. For both scotopic and photopic intensity-response curves, thresholds were defined as the minimal luminance required to reach the criterion amplitude of 10 µV.

### Retinal sections

Animals were sacrificed by a lethal dose of pentobarbital, and their eyes were enucleated, fixed in 4% paraformaldehyde and sequentially cryoprotected in 15, 20 and 30% sucrose. They were then washed in 0.1 M phosphate buffer pH 7.4 (PB), and the cornea, lens and vitreous body were removed. The retinas were then processed for vertical sections. For this purpose, they were embedded in OCT and frozen in liquid N_2_. Sixteen mm-thick sections were then obtained at −25°C, mounted on Superfrost Plus slides (Menzel GmbH & Co KG, Braunschweig, Germany), and air-dried. Prior to subsequent use, slides were thawed and washed 3 times in PB, and then treated with blocking solution (10% normal donkey serum in PB plus 0.5% Triton X-100) for 1 h.

### Retinal immunohistochemistry

To permit objective comparison, retinas from vehicle-treated and safranal-treated rats were fully processed in parallel. Primary antibodies used in this work are summarized in Table 1. Sections were single- or double-immunostained overnight at room temperature with combinations of antibodies against different molecular markers diluted (as indicated in Table 1) in PB containing 0.5% Triton X-100. Alexa Fluor 488 (green)-conjugated anti-rabbit IgG and/or Alexa Fluor 555 (red)-conjugated anti-mouse IgG donkey secondary antibodies from Molecular Probes (Eugene, OR, USA) were then applied at a 1∶100 dilution for 1 h. The sections were finally washed in PB, mounted in Citifluor (Citifluor Ltd; London, UK) and coverslipped for viewing using laser-scanning confocal microscopy on a Leica TCS SP2 system. Immunohistochemical controls were performed by omitting either the primary or secondary antibody. The final images from control and experimental subjects were processed in parallel using Adobe Photoshop 10 software. The thickness of the outer nuclear layer (ONL) was measured by counting the number of photoreceptor rows in retinal sections labeled with the nuclear stain TO-PRO-3 iodide (1∶1000 dilution; Molecular Probes), at distances of 0.5, 1.5, 2.5 and 3.5 mm from the optic nerve, toward both the temporal and nasal ora serratas.

Histochemistry of reduced nicotinamide adenine dinucleotide phosphate diaphorase (NADPH-d) was performed on whole-mount retinas to visualize the retinal vascular network [57]. To accomplish this, retinas were first incubated in a solution of 1 mg/ml NADPH with 0.1 mg/ml of nitroblue tetrazolium (NBT) in 1% Triton X-100 for 1–2 h at 37°C under conditions of darkness. After several washes, the sections were processed for immunocytochemistry using the procedure described above. The morphometrical analysis was performed with the aid of ImageJ software (National Institutes of Health, Bethesda, MD, USA). The relative capillary density was expressed as the ratio between the total capillary area and the retinal surface.

### Statistical analyses

SYSTAT software (London, UK) was used to perform statistical analyses. An ANOVA test was used to evaluate the effects of safranal on ERG responses, and a two-tailed Student's *t*-test was performed to compare the number of rows of photoreceptor cell bodies found in each experimental group. The latter test was also used to evaluate differences between groups in terms of measured capillary density. Normal distributions and homogeneity of variance were found for all analyzed categories. *P* values of less than 0.05 were considered to be statistically significant. Data were plotted as the average ± standard error of the mean (SEM).

## Supporting Information

Figure S1 **Cone morphology in control and safranal-treated P23H animals.** Vertical sections of retinas from a SD rat (A) and P23H rats treated with vehicle (D, F, H) or safranal (B, C, E, G, I) stained with γ-transducin, specific for cone cells. Vehicle-treated P23H animal showed smaller cell size and shorter outer segments and pedicle, as compared to observed in safranal-treated rats. All images were collected from the central area of the retina, close to the optic nerve. ONL: outer nuclear layer, OPL: outer plexiform layer. Scale bar: 20 μm.
(TIF)
