# Supplementary figures and images for: Safranal, a Saffron Constituent, Attenuates Retinal Degeneration in P23H Rats
Source: PLoS One. 2012 Aug 10;7(8):e43074. doi: 10.1371/journal.pone.0043074 (PMC3416780; doi:10.1371/journal.pone.0043074)

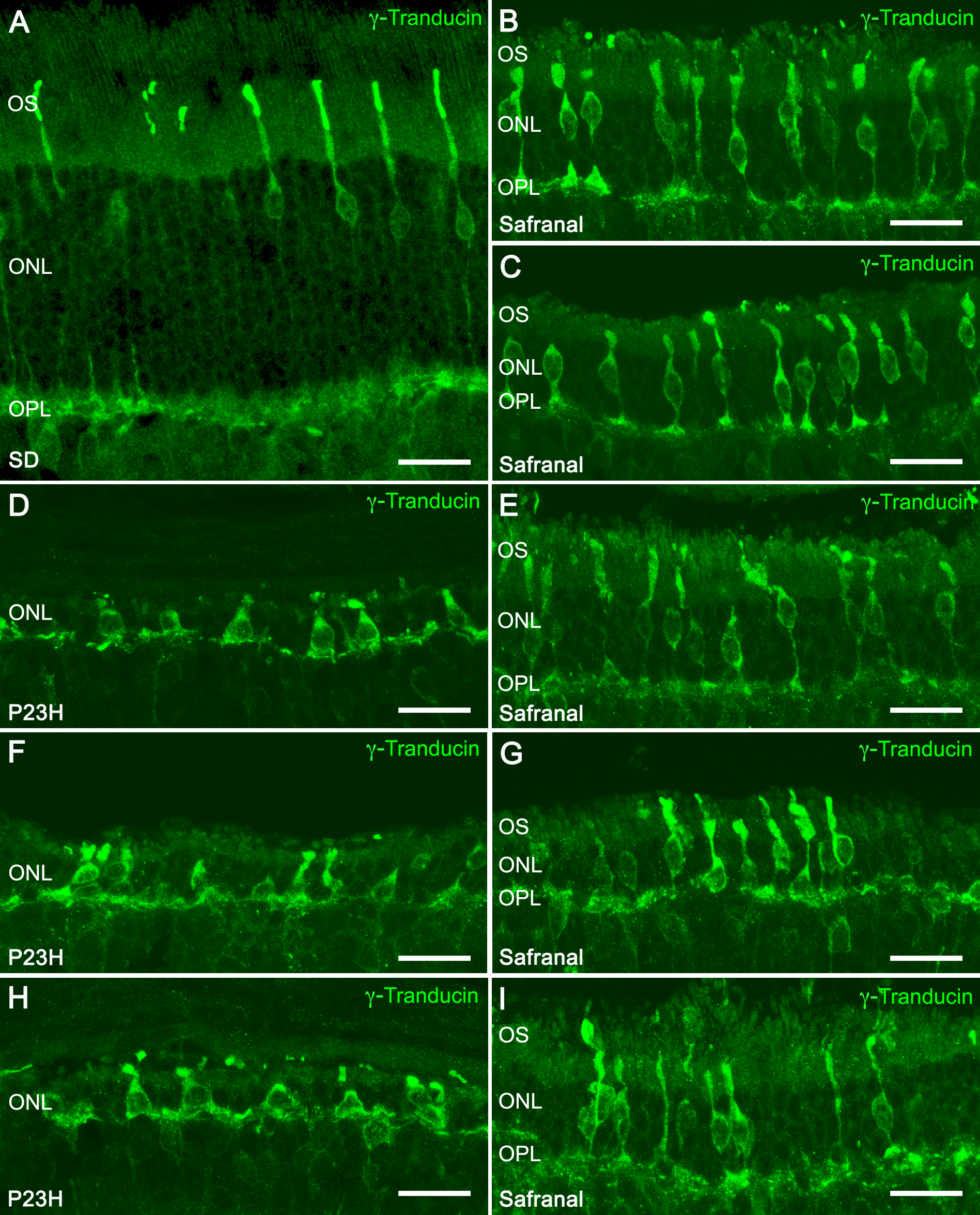

Supplement: Figure S1 — Cone morphology in control and safranal-treated P23H animals. Vertical sections of retinas from a SD rat (A) and P23H rats treated with vehicle (D, F, H) or safranal (B, C, E, G, I) stained with γ-transducin, specific for cone cells. Vehicle-treated P23H animal showed smaller cell size and shorter outer segments and pedicle, as compared to observed in safranal-treated rats. All images were collected from the central area of the retina, close to the optic nerve. ONL: outer nuclear layer, OPL: outer plexiform layer. Scale bar: 20 μm. (TIF) [file pone.0043074.s001.tif]
